# Supplementary material for: A synthetic biochemistry platform for cell free production of monoterpenes from glucose
Source: Nat Commun. 2017 May 24;8:15526. doi: 10.1038/ncomms15526 (PMC5458089; doi:10.1038/ncomms15526)
Supplement: Supplementary Information — Supplementary Figures, Supplementary Tables, Supplementary Note, Supplementary References. [file ncomms15526-s1.pdf]

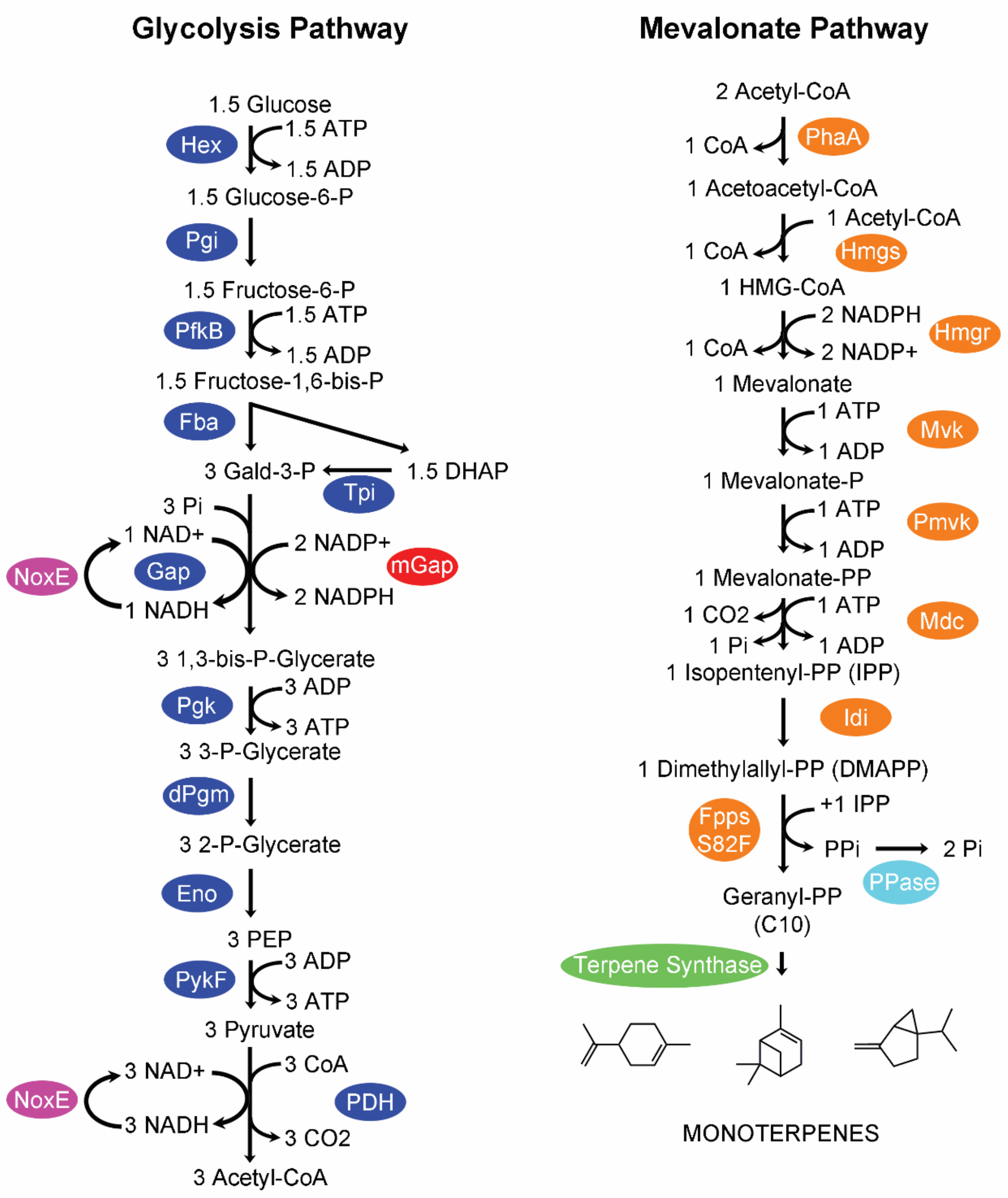

Supplementary Figure 1: Detailed schematic of the synthetic biochemistry system for the conversion of glucose to monoterpenes. Glycolytic and mevalonate enzymes are highlighted in blue and orange respectively. Auxiliary enzymes constituting the purge valve, (mGap and NoxE) and phosphate recycle by pyrophosphatase are highlighted in red, purple, and cyan. Different monoterpenes can be made by using alternative terpene synthases (highlighted green). Enzymes) are labeled as described in the text and Supplementary Table 1.

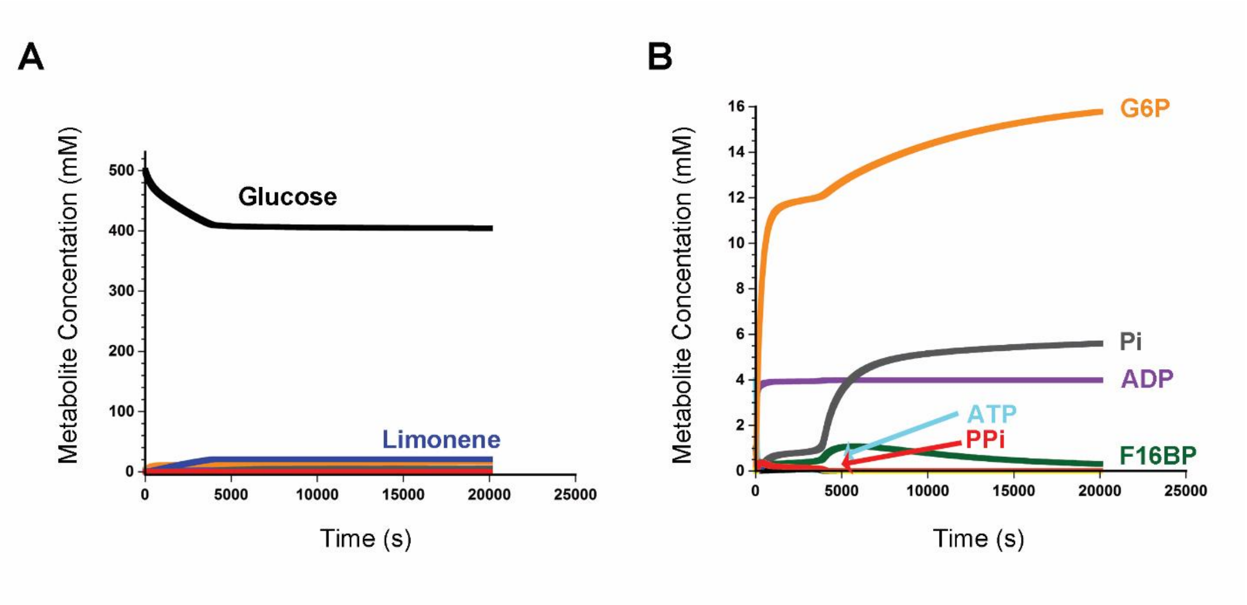

Supplementary Figure 2: Metabolite levels from CoPASI for unbalanced system. (A) Levels for glucose (black line) and limonene (blue line) in an unbalanced CoPASI model (high Hex) starting at 500 mM glucose and a time of 20000 seconds (5.5 hours). (B) Expanded view of (A) showing levels of Pi, Glucose-6-phosphate (G6P), fructose-1,6-bisphosphate (F16BP) and ADP.

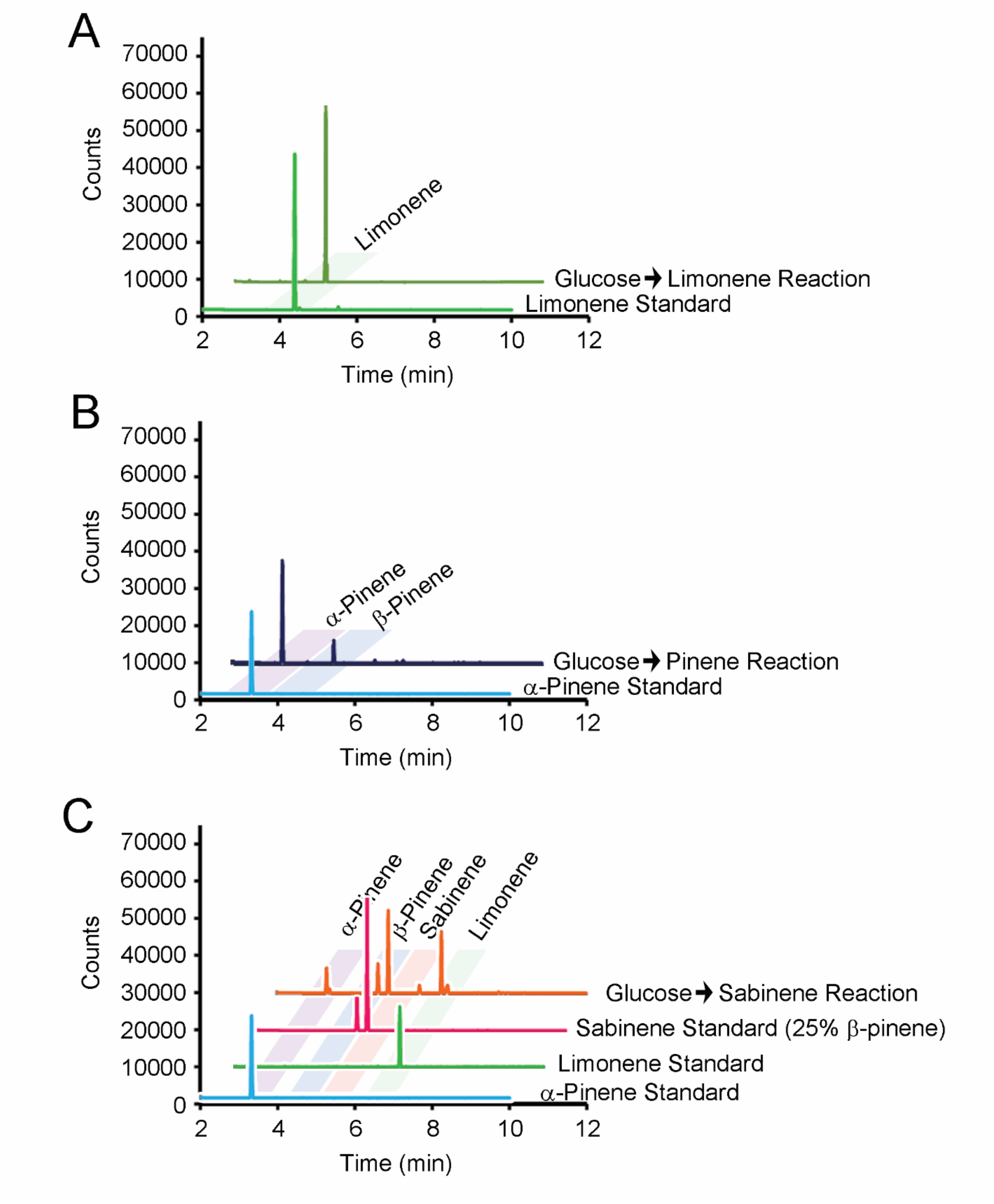

Supplementary Figure 3: GC analysis of glucose to monoterpene reactions. (A) Representative trace of limonene production starting from 500 mM glucose after 7 days using *M. spicata* limonene synthase (B) Representative trace of pinene production starting from 500 mM glucose after 7 days using *P. sitchensis* α-pinene synthase. (C) Representative trace of sabinene production starting from 500 mM glucose after 7 days using *M. spicata* limonene synthase mutant N345A.

Supplementary Table 1: Enzyme Table

	Enzyme	*Organism*	Source	Vector	Tag	Accession Number	Ref.
Hex	Hexokinase	*S. cerevisiae*	Sigma				
Pgi	Glucose-6-phosphate isomerase	*G. thermodenitrificans*	genomic	pET28	N-His	ABO68222	
Pfk	Phosphofructokinase	*G. stearothermophilus*	genomic	pET28	N-His	KOR92562	
Fba	Fruc-1,6-BP aldolase	*S. aureus*	genomic	pET28	N-His	BAR10119	1
Tpi	Triose Phosphate Isomerase	*G. stearothermophilus*	genomic	pET28	N-His	KOR95273	
Gap	Gald-3-P Dehydrogenase	*E. coli K12*	genomic	pET28	N-His	NP_416293	
mGap	Gald-3-P Dehydrogenase-D34A/L35R/T36K	*G. stearothermophilus*	genomic	pET28	N-His	KOR95271	2
Pgk	Phosphoglycerate Kinase	*G. stearothermophilus*	genomic	pET28	N-His	KOR95272	
Gpm	Phosphoglycerate Mutase (2,3-BPG dependent)	*E. coli K12*	genomic	pET28	N-His	NP_415276	
Eno	PEP hydratase	*E. coli K12*	genomic	pET28	N-His	NP_417259	
Pyk	Pyruvate Kinase	*E. coli K12*	genomic	pET28	N-His	NP_416191	
Pdh	Pyruvate Dehydrogenase Complex	*E. coli K12*					
	pyruvate decarboxylase; E1 (AceE)	*E. coli K12*	genomic	pET28	N-His	NP_414656	This study
	dihydrolipoyltransacetylase; E2 (AceF)	*E. coli K12*	genomic	pET28	N-His	NP_414657	This study
	dihydrolipoyl dehydrogenase; E3 (Lpd)	*E. coli K12*	genomic	pET28	N-His	NP_414658	This study
Thl	Acetyl-CoA acetyltransferase	*R. eutropha H16*	genomic	pET28	N-His	CAJ92573	
Hmgs	HMG-CoA synthase-A110G	*E. faecalis*	genomic	pET28	N-His	WP_010785222	3
Hmgr	HMG-CoA reductase	*E. faecalis*	genomic	pET28	N-His	AAG02439	
Mvk	Mevalonate kinase	*M. mazei*	genomic	pET28	N-His	AAM31458	
Pmvk	Phosphomevalonate kinase	*S. pneumoniae*	genomic	pET28	N-His	WP_000562411	
Mdc	Mevalonate-PP decarboxylase	*S. pneumoniae*	genomic	pET28	N-His	NP_357933	
Idi	Isopentenyl-PP isomerase	*E. coli K12*	genomic	pET28	C-His	NP_417365	
Fpps-S82F	Farnesyl-PP synthase-S82F mutant	*G. stearothermophilus*	genomic	pET28	N-His	KOR95521	4
LimSyn	Limonene synthase	*M. spicata*	Addgene	pET28	N-His	AAC37366	
LimSyn-N345A	Limonene synthase-N345A mutant	*M. spicata*		pET28	N-His	AAC37366	5
PinSyn	α-Pinene synthase (*Q83)	*P. sitchensis*	iGem	pET28-SUMO	N-His-SUMO	ADZ45509	
NoxE	NADH oxidase	*L. lactis*	genomic	pET22	C-His	WP_015425842	
PPase	Pyrophosphatase	*G. stearothermophilus*	genomic	pET28	N-His	KOR95080	
Gor	Glutathione reductase	*E. coli K12*	ASKA	pCA24N	N-His	NP_417957	
Cat	Catalase	*C. glutamicum*	Sigma				

Supplementary Table 2: CoPASI Modelling Parameters

	Enzyme[#]	U (V_{max})	K_m (forward)	K_m (reverse)	K_{eq}	Metabolite/ Cofactor	Start Conc. (mM)
1	Hex	0.05	ATP: 0.1<br>Glc: 0.15	ADP: 0.1<br>G6P: 0.1	1000	Glucose	500
2	Pgi	1	G6P: 0.3	F6P: 0.15	0.5	G6P	0
3	Pfk	0.5	ATP: 0.1<br>F6P: 0.03	ADP: 0.1<br>FBP: 0.1	300	F6P	0
4	Fba	1	FBP: 0.015	DHAP: 0.1<br>G3P: 0.1	0.2	F16BP	1
5	Tpi	0.25	DHAP: 1	G3P: 1	0.04	DHAP	0
6	Gap	0.5	G3P: 1<br>NAD: 0.05<br>Pi: 0.5	1,3BPG: 2<br>NADH: 0.1	0.07	Gald3P	0
7	mGap	10	G3P: 1<br>NADP: 0.05<br>Pi: 0.5	1,3BPG: 2<br>NADPH: 0.1	0.07	DHAP	0
8	Pgk	2	1,3BPG: 0.1<br>ADP: 0.1	3PG: 2.2<br>ATP: 3	3200	1,3BPG	0
9	Pgm	1	3PG: 0.5	2PG: 0.1	0.19	3PG	0
10	Eno	1	2PG: 0.1	PEP: 1	6.7	2PG	0
11	Pyk	1.0	PEP: 0.3<br>ADP: 0.3	PYR: 1<br>ATP: 1	5000	PEP	0
12	Pdh	10	CoA: 0.1<br>NAD: 0.1<br>PYR: 0.5	AcAoA: 0.1<br>NADH: 0.1<br>CO2: 0.1	5000	PYR	0
13	PhaA	1	AcCoA: 0.4	AcAcCoA: 0.1<br>CoA: 0.1	0.05	ACCoA	0
14	Hmgs	1	AcCoA: 0.4<br>AcAcCoA: 0.01	HMGCoA: 0.5<br>CoA: 0.5	50	ACACCoA	0
15	Hmgr	1	HMGCoA: 0.02<br>NADPH: 0.1	MEV: 1<br>NADP: 0.3<br>CoA: 0.3	50	HMGCoA	0
16	Mvk	5	MEV: 0.07<br>ATP: 0.5	MEVP: 1.5<br>ADP: 0.1	10	MEV	0
17	Pmvk	5	MEVP: 0.008<br>ATP: 0.14	MEVPP: 0.01<br>ADP: 0. 4	2	MEVP	0
18	Mdc	5	MEVPP: 0.1<br>ATP: 0.06	IPP: 0.5<br>ADP: 0.3<br>CO2: 5<br>Pi: 0.5	10	MEVPP	0
19	Idi	1	IPP: 0.004	DMAPP: 0.05	0.77	IPP	0
20	Fpps-S82F	1	IPP: 0.005<br>DMAPP: 0.005	GPP: 4<br>PPi: 5	200	DMAPP	0
21	LimSyn	5	GPP: 0.002	LIM: 2<br>PPi: 0.5	5000	GPP	0
22	PPase	20	PPi: 0.1	Pi: 0.1	10	LIM	0
23	NoxE	1	NADH: 0.1			Pi	25
						PPi	0
						NAD+	0.25
						NADP+	1.5
						ATP	4
						ADP	0
						CoA	1.5

# - Refer to Supplemental Table 1 for enzyme origin

Supplementary Table 3: Enzyme Activity and Units Added

	Enzyme#	mg mL^{-1}	mg added	Units(U) mg^{-1}	U added	U (ratio to Hex)	Total Turnovers
1	Hex	9.6	0.001	7.2 ± 0.1	0.01	1.0	2.6 (3.3) x 10^6
2	Pgi	5.3	0.03	19.2 ± 0.2	0.6	85.3	0.9 (1.2) x 10^5
3	Pfk	8.1	0.02	9.6 ± 0.2	0.2	32.0	8.2 (10.5) x 10^4
4	Fba	13.6	0.04	21.1 ± 0.3	0.9	120.2	4.8 (6.1) x 10^4
5	Tpi	9.5	0.004	96.6 ± 5.4	0.4	53.7	4.3 (5.4) x 10^5
6	Gap	13.6	0.007	9.3 ± 0.7	0.1	9.0	6.1 (7.8) x 10^5
7	mGap	6.8	0.03	12.9 ± 0.6	0.4	60.9	1.2 (1.6) x 10^5
8	Pgk	11.4	0.01	102.3 ± 2.9	1.1	156.3	4.3 (5.5) x 10^5
9	Pgm	11.8	0.05	97.2 ± 13.0	4.6	634.5	7.2 (9.1) x 10^4
10	Eno	17.3	0.05	82.5 ± 0.1	1.4	194.8	3.0 (3.9) x 10^5
11	Pyk	2.0	0.01	365.4 ± 37.9	2.9	406.0	7.3 (9.3) x 10^5
12	Pdh	9.9	0.01	1.4 ± 0.2	0.2	19.3	6.8 (8.6) x 10^6
13	PhaA	7.9	0.02	81.1 ± 5.7	1.3	180.2	1.5 (1.9) x 10^5
14	Hmgs	5.8	0.03	1.5 ± 0.1	0.04	6.0	5.6 (7.1) x 10^4
15	Hmgr	16.6	0.08	4.2 ± 0.4	0.4	48.4	2.1 (2.7) x 10^4
16	Mvk	4.7	0.02	8.1 ± 0.6	0.2	27.0	5.2 (6.7) x 10^4
17	Pmvk	7.8	0.04	14.8 ± 0.1	0.6	80.2	3.7 (4.7) x 10^4
18	Mdc	7.9	0.08	4.1 ± 0.1	0.3	45.0	1.9 (2.4) x 10^4
19	Idi	14.2	0.07	4.3	0.3	42.4	1.2 (1.5) x 10^4
20	Fpps-S82F	19.2	0.02	7.4	0.1	19.5	3.3 (4.2) x 10^4
21	LimSyn	28.6	0.6	0.01	0.0		2310
22	LimSyn-N345A	28.4	0.6	0.01	0.0		2940
23	PinSyn	19.3	0.4	0.01	0.004		4310
24	NoxE	9.7	0.05	43.4 ± 2.5	2.1	295.4	2.5 (3.2) x 10^4
25	PPase	9.9	0.02	4.4 ± 0.5	0.1	12.1	2.0 (2.5) x 10^4
26	GorA	5.7	0.01	32.1 ± 0.1	0.2		-
27	Catalase	4.8	0.001	104 x 10^6	104.2		-
	Total mg - 1.3 (0.8$) Total mg/mL - 6.6 (3.8$)						Average: 5.5 (7.0) x 10^5

# - Refer to Supplemental Table 1 for enzyme origin
* - Units expressed as μmol/min/mg of enzyme
$ - without terpene synthase

Supplementary Table 4: Primers Used for Cloning

	Enzyme	Primer Sequence (F: Forward Primer, R: Reverse Primer, M: Mutagenic Primer)
1	Hex	From sigma
2	Pgi	F: 5’ GGTGCCGCGCGGCAGCCATATGACCCATATTCGTTTCGAC 3’<br>R: 5’ CAGTGGTGGTGGTGGTGGTGCTCGAGTTATTTCAACCGTTTCTCCAGTTC 3’
3	Pfk	F: 5’ CCTGGTGCCGCGCGGCAGCCATATGAAACGCATTGGTGTGTTG 3’<br>R: 5’ CAGTGGTGGTGGTGGTGGTGCTCGAGTTAAATGGACAATTCTTTCGAC 3’
4	Fba	F: 5’ GCCGCGCGGCAGCCATATGAATAAAGAGCAATTAGAAAAAATGAAAAATGGAAAAGGC 3’<br>R: 5’ GGTGGTGGTGGTGGTGCTCGAGTTAGTTTTTGTTTACAGATGCGTCGTAG 3’
5	Tpi	F: 5’ GGTGCCGCGCGGCAGCCATATGAGAAAACCGATCATTGCAGG 3’<br>R: 5’ GGTGGTGGTGGTGGTGCTCGAGTTACTCATGACGCCCCCGC 3’
6	Gap	F: 5’ GGTGCCGCGCGGCAGCCATATGACTATCAAAGTAGGTATCAACGGTTTTGGCC 3’<br>R: 5’ GGTGGTGGTGGTGGTGCTCGAGTTATTTGGAGATGTGAGCGATCAGGTCCAG 3’
7	mGap	F: 5’ CCTGGTGCCGCGCGGCAGCCATATGGCAGTCAAAGTGGGAATCAAC 3’<br>R: 5’ CAGTGGTGGTGGTGGTGGTGCTCGAGTTACAGCCCTTTCGAGGCGGTG 3’<br>M: 5’ TGGTGGCGGTGAACGCGCGTAAAGATGCGAATACGCTT 3’
8	Pgk	F: 5’ GGTGCCGCGCGGCAGCCATATGAACAAGAAGACGATCCGC 3’<br>R: 5’ GGTGGTGGTGGTGGTGCTCGAGTTATTTGTCTTCGAGTGCGACG 3’
9	Pgm	ASKA Clone
10	Eno	F: 5’ GGTGCCGCGCGGCAGCCATATGTCCAAAATCGTAAAAATCATCGGTCGTGAAATC 3’<br>R: 5’ GGTGGTGGTGGTGGTGCTCGAGTTATGCCTGGCCTTTGATCTCTTTACGACC 3’
11	Pyk	F: 5’ GGTGCCGCGCGGCAGCCATATGAAAAAGACCAAAATTGTTTGCACCATCGGACC 3’<br>R: 5’ CAGTGGTGGTGGTGGTGGTGCTCGAGTTACAGGACGTGAACAGATGCGGTG 3’
12a	AceE	F: 5’ GGTGCCGCGCGGCAGCCATATGTCAGAACGTTTCCCAAATGACGTGG 3’<br>R: 5’ GGTGGTGGTGGTGGTGCTCGAGTTACGCCAGACGCGGGTTAACTTTATC 3’
12b	AceF	F: 5’ GGTGCCGCGCGGCAGCCATATGGCTATCGAAATCAAAGTACCGGACATCG 3’<br>R: 5’ GGTGGTGGTGGTGGTGCTCGAGTTACATCACCAGACGGCGAATGTCAGAC 3’
12c	Lpd	F: 5’GGTGCCGCGCGGCAGCCATATGAGTACTGAAATCAAAACTCAGGTCGTGGTACTTGG 3’<br>R: 5’ GGTGGTGGTGGTGGTGCTCGAGTTACTTCTTCTTCGCTTTCGGGTTCGGC 3’
13	PhaA	F: 5’ CCTGGTGCCGCGCGGCAGCCATATGATGACTGACGTTGTCATCGTATCCGCC 3’<br>R: 5’ CGGCCGCAAGCTTGTCGACGGAGCTTTATTTGCGCTCGACTGCCAGCGCCACG 3’
14	Hmgs-A110G	F: 5’ CCTGGTGCCGCGCGGCAGCCATATGACAATTGGGATTGATAAAATTAGTTTTTTTGTGC 3’<br>R: 5’ GTGGTGGTGCTCGAGTTAGTTTCGATAAGAGCGAACGGTATTATTAATAGC 3’<br>M: 5’ AAGGAAGGTTGTTACGGAGCAA 3’
15	Hmgr	F: 5’ GGTGCCGCGCGGCAGCCATATGGAGAGACCTCAGCAAAAAAAAAACAGCCGATTTTATC 3’<br>R: 5’ GTGGTGGTGGTGGTGCTCGAGTTATTGTTTTCTTAAATCATTTAAAATAGCCAAGGC 3’
16	Mvk	F: 5’ GGTGCCGCGCGGCAGCCATATGGTTTCATGTTCTGCGCCCG 3’<br>R: 5’ CAGTGGTGGTGGTGGTGGTGCTCGAGTCAATCGACCTTCAACCCCTGTTC 3’
17	Pmvk	F: 5’ GCCGCGCGGCAGCCATATGATTGCTGTTAAAACTTGCGGAAAACTCTATTGG 3’<br>R: 5’ GGTGGTGGTGGTGGTGCTCGAGTTATGATTTGTCGTCATGTCCTATCCTTTCTTG 3’
18	Mdc	F: 5’ GCCGCGCGGCAGCCATATGGATAGAGAGCCTGTGACAGTACGTTCC 3’<br>R: 5’ GGTGGTGGTGGTGGTGCTCGAGTTAACAGCAATCATCTTGACTCAAATCCTTTGTTTTTG 3’
19	Idi	F: 5’ GGAGATATACATATGCAAACGGAACACGTCATTTTATTGAATG 3’<br>R: 5’ GTGGTGGTGGTGCTCGAGTTTAAGCTGGGTAAATGCAGATAATCG 3’
20	Fpps-S82F	F: 5’ GGTGCCGCGCGGCAGCCATATGGCGCAGCTTTCAGTTGAACAG 3’<br>R: 5’ CAGTGGTGGTGGTGGTGGTGCTCGAGTTAATGGTCGCGGGCGGC 3’<br>M: 5’ GATCCATACGTACTTTTTGATCCATGATG 3’
21	LimSyn	F: 5’ GCCTGGTGCCGCGCGGCAGCCATATGATGCGTCGCAGTGGTAATTACAACCCG 3’<br>R: 5’ GGCCGCAAGCTTGTCGACGGAGCTCTTAGGCGAAAGGTGCAAACAGGGTACGCG 3’
22	LimSyn-N345A	M: 5’ GATGGGTAAGGTCGCGGCGCTGATTACTG 3’
23	PinSyn	F: 5’ GAGAACAGATTGGTGGATCCCAAAGACGGATGGGCGATTTCCATTCCAACC 3’<br>R: 5’ GTGGTGGTGCTCGAGTTACAAAGTCACAGGATCAATCACGGTTCTCTTC 3’
24	NoxE	F: 5’ GGAGATATACATATGAAAAATCGTAGTTATCGGTACGAACCACGCAGGCATTGC 3’<br>R: 5’ GGTGGTGGTGGTGGTGCTCGAGTTTTGCATTTAAAGCTGCAACAGTCATGTAATTATATGG 3’
25	PPase	F: 5’ GCCGCGCGGCAGCCATATGGCCTTTGAGAATAAGATTGTCGAAGCGTTTATCG 3’<br>R: 5’ GGTGGTGGTGGTGGTGCTCGAGTTATTTTTGTTCGTTATAGCGGGCGATGCACTC 3’
26	GorA	ASKA Clone
27	Catalase	From Sigma

Supplementary Note

The following Supplementary equations 1-23 were used to model the production of limonene from glucose using the described metabolic pathway consisting of modified glycolysis, pyruvate dehydrogenase, and the mevalonate pathway. The equations were derived based off of established rate equations used to model yeast glycolysis. Implementing these equations in CoPASI in combination with starting levels of metabolites described in Supplementary Table 2 allows limonene production from glucose to be modelled over time.

Rate Law for Hexokinase (Hex)

$$\mathrm{Vmax} * \frac{\frac{\mathrm{A} * \mathrm{B} - \mathrm{P} * \frac{\mathrm{Q}}{\mathrm{Keq}}}{\mathrm{Katp} * \mathrm{Kglc}}}{\left(1 + \frac{\mathrm{A}}{\mathrm{Katp}}\right) * \left(1 + \frac{\mathrm{B}}{\mathrm{Kglc}}\right) + \left(1 + \frac{\mathrm{P}}{\mathrm{Kadp}}\right) * \left(1 + \frac{\mathrm{Q}}{\mathrm{Kg6p}}\right) - 1} \tag{1}$$

Rate Law for Phosphoglucoseisomerase (Pgi)

$$\mathrm{Vmax} * \frac{\frac{\mathrm{A} - \frac{\mathrm{P}}{\mathrm{Keq}}}{\mathrm{Kg6p}}}{1 + \frac{\mathrm{A}}{\mathrm{Kg6p}} + 1 + \frac{\mathrm{P}}{\mathrm{Kf6p}} - 1} \tag{2}$$

Rate Law for Phosphofructokinase (Pfk)

$$\mathrm{Vmax} * \frac{\frac{\mathrm{A} * \mathrm{B} - \mathrm{P} * \frac{\mathrm{Q}}{\mathrm{Keq}}}{\mathrm{Katp} * \mathrm{Kf6p}}}{\left(1 + \frac{\mathrm{A}}{\mathrm{Katp}}\right) * \left(1 + \frac{\mathrm{B}}{\mathrm{Kf6p}}\right) + \left(1 + \frac{\mathrm{P}}{\mathrm{Kadp}}\right) * \left(1 + \frac{\mathrm{Q}}{\mathrm{Kfbp}}\right) - 1} \tag{3}$$

Rate Law for Fructose-1,6-Bisphosphate Aldolase (Fba)

$$\mathrm{Vmax} * \frac{\frac{\mathrm{A} - \mathrm{P} * \frac{\mathrm{Q}}{\mathrm{Keq}}}{\mathrm{Kfbp}}}{\left(1 + \frac{\mathrm{A}}{\mathrm{Kfbp}}\right) + \left(1 + \frac{\mathrm{P}}{\mathrm{Kdhap}}\right) * \left(1 + \frac{\mathrm{Q}}{\mathrm{Kgap}}\right) - 1} \tag{4}$$

Rate Law for Triosephosphate Isomerase (Tpi)

$$\mathrm{Vmax} * \frac{\frac{\mathrm{A} - \frac{\mathrm{P}}{\mathrm{Keq}}}{\mathrm{Kdhap}}}{1 + \frac{\mathrm{A}}{\mathrm{Kdhap}} + 1 + \frac{\mathrm{P}}{\mathrm{Kgap}} - 1} \tag{5}$$

Rate Law for Glyceraldehyde-3-Phosphate Dehydrogenase (Gap)

$$\mathrm{Vmax} * \frac{\frac{\mathrm{A} * \mathrm{B} * \mathrm{C} - \mathrm{P} * \frac{\mathrm{Q}}{\mathrm{Keq}}}{\mathrm{Kgap} * \mathrm{Knad} * \mathrm{Kppi}}}{\left(1 + \frac{\mathrm{A}}{\mathrm{Kgap}}\right) * \left(1 + \frac{\mathrm{B}}{\mathrm{Knad}}\right) * \left(1 + \frac{\mathrm{C}}{\mathrm{Kppi}}\right) + \left(\frac{\mathrm{P}}{\mathrm{Kbpg}}\right) * \left(1 + \frac{\mathrm{Q}}{\mathrm{Knadh}}\right) - 1} \tag{6}$$

Rate Law for Mutant Glyceraldehyde-3-Phosphate Dehydrogenase (mGap)

$$\mathrm{Vmax} * \frac{\frac{\mathrm{A} * \mathrm{B} * \mathrm{C} - \mathrm{P} * \frac{\mathrm{Q}}{\mathrm{Keq}}}{\mathrm{Kgap} * \mathrm{Knadp} * \mathrm{Kppi}}}{\left(1 + \frac{\mathrm{A}}{\mathrm{Kgap}}\right) * \left(1 + \frac{\mathrm{B}}{\mathrm{Knadp}}\right) * \left(1 + \frac{\mathrm{C}}{\mathrm{Kppi}}\right) + \left(\frac{\mathrm{P}}{\mathrm{Kbpg}}\right) * \left(1 + \frac{\mathrm{Q}}{\mathrm{Knadph}}\right) - 1} \tag{7}$$

Rate Law for Phosphoglycerate Kinase (Pgk)

$$\mathrm{Vmax} * \frac{\frac{A * B - P * \frac{Q}{\mathrm{Keq}}}{\mathrm{Kbpg} * \mathrm{Kadp}}}{\left(1 + \frac{A}{\mathrm{Kbpg}}\right) * \left(1 + \frac{B}{\mathrm{Kadp}}\right) + \left(1 + \frac{P}{\mathrm{Kp3g}}\right) * \left(1 + \frac{Q}{\mathrm{Katp}}\right) - 1} \tag{8}$$

Rate Law for Phosphoglycerate Mutase (Pgm)

$$\mathrm{Vmax} * \frac{\frac{A - \frac{P}{\mathrm{Keq}}}{\mathrm{Kp3g}}}{1 + \frac{A}{\mathrm{Kp3g}} + 1 + \frac{P}{\mathrm{Kp2g}} - 1} \tag{9}$$

Rate Law for Enolase (Eno)

$$\mathrm{Vmax} * \frac{\frac{A - \frac{P}{\mathrm{Keq}}}{\mathrm{Kp2g}}}{1 + \frac{A}{\mathrm{Kp2g}} + 1 + \frac{P}{\mathrm{Kpep}} - 1} \tag{10}$$

Rate Law for Pyruvate Kinase (Pyk)

$$\mathrm{Vmax} * \frac{\frac{A * B - P * \frac{Q}{\mathrm{Keq}}}{\mathrm{Kadp} * \mathrm{Kpep}}}{\left(1 + \frac{A}{\mathrm{Kadp}}\right) * \left(1 + \frac{B}{\mathrm{Kpep}}\right) + \left(1 + \frac{P}{\mathrm{Katp}}\right) * \left(1 + \frac{Q}{\mathrm{KPYR}}\right) - 1} \tag{11}$$

Rate Law for Pyruvate Dehydrogenase (Pdh)

$$\mathrm{Vmax} * \frac{\frac{A * B * C - \left(P * Q * \frac{Z}{\mathrm{Keq}}\right)}{\mathrm{KCOA} * \mathrm{Knad} * \mathrm{KPYR}}}{\left(1 + \frac{A}{\mathrm{KCOA}}\right) * \left(1 + \frac{B}{\mathrm{Knad}}\right) * \left(1 + \frac{C}{\mathrm{KPYR}}\right) + \left(1 + \frac{P}{\mathrm{KACCOA}}\right) * \left(1 + \frac{Q}{\mathrm{KCO2}}\right) * \left(1 + \frac{Z}{\mathrm{Knadh}}\right) - 1} \tag{12}$$

Rate Law for Thiolase (Thl)

$$\mathrm{Vmax} * \frac{\frac{(A)^2 - P * \frac{Q}{\mathrm{Keq}}}{(\mathrm{KACCOA})^2}}{\left(1 + \frac{A}{\mathrm{KACCOA}}\right)^2 + \left(1 + \frac{P}{\mathrm{KACACCOA}}\right) + \left(1 + \frac{Q}{\mathrm{KCOA}}\right) - 1} \tag{13}$$

Rate Law for HMG-CoA Synthase (Hmgs)

$$\mathrm{Vmax} * \frac{\frac{A * B - P * \frac{Q}{\mathrm{Keq}}}{\mathrm{KACACCOA} * \mathrm{KACCOA}}}{\left(1 + \frac{A}{\mathrm{KACACCOA}}\right) * \left(1 + \frac{B}{\mathrm{KACCOA}}\right) + \left(1 + \frac{P}{\mathrm{KHMGCOA}}\right) * \left(1 + \frac{Q}{\mathrm{KCOA}}\right) - 1} \tag{14}$$

Rate Law for HMG-CoA Reductase (Hmgr)

$$\mathrm{Vmax} * \frac{\frac{A * (B)^2 - P * Q * \frac{(Z)^2}{\mathrm{Keq}}}{\mathrm{KHMGCOA} * (\mathrm{Knadph})^2}}{\left(1 + \frac{A}{\mathrm{KHMGCOA}}\right) * \left(1 + \frac{B}{\mathrm{Knadph}}\right)^2 + \left(1 + \frac{P}{\mathrm{Kmev}}\right) * \left(1 + \frac{Q}{\mathrm{KCOA}}\right) * \left(1 + \frac{Z}{\mathrm{Knadp}}\right)^2 - 1} \tag{15}$$

Rate Law for Mevalonate Kinase (Mvk)

$$\mathrm{Vmax} * \frac{\mathrm{A} * \frac{\mathrm{B}}{\mathrm{Kmev} * \mathrm{Katp}} - \mathrm{P} * \frac{\mathrm{Q}}{\mathrm{Kmev} * \mathrm{Katp} * \mathrm{Keq}}}{\left(1 + \frac{\mathrm{A}}{\mathrm{Kmev}} + \frac{\mathrm{P}}{\mathrm{Kmvp}}\right) * \left(1 + \frac{\mathrm{B}}{\mathrm{Katp}} + \frac{\mathrm{Q}}{\mathrm{Kadp}}\right)} \tag{16}$$

Rate Law for Phosphomevalonate Kinase (Pmvk)

$$\mathrm{Vmax} * \frac{\mathrm{A} * \frac{\mathrm{B}}{\mathrm{Kmvp} * \mathrm{Katp}} - \mathrm{P} * \frac{\mathrm{Q}}{\mathrm{Kmvp} * \mathrm{Katp} * \mathrm{Keq}}}{\left(1 + \frac{\mathrm{A}}{\mathrm{Kmvp}} + \frac{\mathrm{P}}{\mathrm{Kmpp}}\right) * \left(1 + \frac{\mathrm{B}}{\mathrm{Katp}} + \frac{\mathrm{Q}}{\mathrm{Kadp}}\right)} \tag{17}$$

Rate Law for Mevalonate Pyrophosphate Decarboxylase (Mdc)

$$\mathrm{Vmax} * \frac{\frac{\mathrm{A} * \mathrm{B} - \left(\mathrm{P} * \mathrm{Q} * \mathrm{Z} * \frac{\mathrm{X}}{\mathrm{Keq}}\right)}{\mathrm{Kmpp} * \mathrm{Katp}}}{\left(1 + \frac{\mathrm{A}}{\mathrm{Kmpp}}\right) * \left(1 + \frac{\mathrm{B}}{\mathrm{Katp}}\right) + \left(1 + \frac{\mathrm{P}}{\mathrm{Kadp}}\right) * \left(1 + \frac{\mathrm{Q}}{\mathrm{KCO2}}\right) * \left(1 + \frac{\mathrm{Z}}{\mathrm{Kipp}}\right) * \left(1 + \frac{\mathrm{X}}{\mathrm{Kppi}}\right) - 1} \tag{18}$$

Rate Law for Isopentenyl Diphosphate Isomerase (Idi)

$$\mathrm{Vmax} * \frac{\frac{\mathrm{A} - \left(\frac{\mathrm{P}}{\mathrm{Keq}}\right)}{\mathrm{Kipp}}}{\left(1 + \frac{\mathrm{A}}{\mathrm{Kipp}}\right) + \left(1 + \frac{\mathrm{P}}{\mathrm{Kdmapp}}\right) - 1} \tag{19}$$

Rate Law for Farnesyl Pyrophosphate Synthase-S82F (Fpps-S82F)

$$\mathrm{Vmax} * \frac{\frac{\mathrm{A} * \mathrm{B} - \mathrm{P} * \frac{\mathrm{Q}}{\mathrm{Keq}}}{\mathrm{Kdmapp} * \mathrm{Kipp}}}{\left(1 + \frac{\mathrm{A}}{\mathrm{Kdmapp}}\right) * \left(1 + \frac{\mathrm{B}}{\mathrm{Kipp}}\right) + \left(1 + \frac{\mathrm{P}}{\mathrm{Kgpp}}\right) * \left(1 + \frac{\mathrm{Q}}{\mathrm{Kpppi}}\right) - 1} \tag{20}$$

Rate Law for Limonene Synthase (Lim)

$$\mathrm{Vmax} * \frac{\frac{\mathrm{A} - \mathrm{P} * \frac{\mathrm{Q}}{\mathrm{Keq}}}{\mathrm{KGPP}}}{1 + \frac{\mathrm{A}}{\mathrm{KGPP}} + \left(1 + \frac{\mathrm{P}}{\mathrm{KLIM}}\right) * \left(1 + \frac{\mathrm{Q}}{\mathrm{Kpppi}}\right) - 1} \tag{21}$$

Rate Law for Pyrophosphatase (PPase)

$$\mathrm{Vmax} * \frac{\frac{\mathrm{A} - \frac{(\mathrm{P})^2}{\mathrm{Keq}}}{\mathrm{Kpppi}}}{\left(1 + \frac{\mathrm{A}}{\mathrm{Kpppi}}\right) + \left(1 + \frac{\mathrm{P}}{\mathrm{Kppi}}\right)^2 - 1} \tag{22}$$

Rate Law for NADH oxicase (NoxE)

$$\mathrm{Vmax} * \frac{\mathrm{A}}{\mathrm{Knadh} + \mathrm{A}} \tag{23}$$

Supplementary References

1. Götz, F., Fischer, S. & Schleifer, K.-H. Purification and Characterisation of an Unusually Heat-Stable and Acid/Base-Stable Class I Fructose-1,6-bisphosphate Aldolase from Staphylococcus aureus. *Eur. J. Biochem.* **108,** 295–301 (1980).
2. Opgenorth, P. H., Korman, T. P. & Bowie, J. U. A molecular rheostat design that maintains ATP levels needed to drive cell-free synthetic biochemistry systems. *Submitted* (2016).
3. Steussy, C. N. *et al.* A Structural Limitation on Enzyme Activity: The Case of HMG-CoA Synthase†,‡. *Biochemistry (Mosc.)* **45,** 14407–14414 (2006).
4. Narita, K., Ohnuma, S. & Nishino, T. Protein Design of Geranyl Diphosphate Synthase. Strutural Features That Define the Product Specificities of Prenyltransferases. *J. Biochem. (Tokyo)* **126,** 566–571 (1999).
5. Srividya, N., Davis, E. M., Croteau, R. B. & Lange, B. M. Functional analysis of (4S)-limonene synthase mutants reveals determinants of catalytic outcome in a model monoterpene synthase. *Proc. Natl. Acad. Sci.* **112,** 3332–3337 (2015).
